# Does anthocyanins consumption affect weight and body composition? A systematic review and meta‐analysis of randomized controlled trials

**DOI:** 10.1002/osp4.651

**Published:** 2023-01-23

**Authors:** Faezeh Yarhosseini, Mina Darand, Zohreh Sadat Sangsefidi, Hassan Mozaffari‐Khosravi, Mahdieh Hosseinzadeh

**Affiliations:** ^1^ Nutrition and Food Security Research Center Shahid Sadoughi University of Medical Sciences Yazd Iran; ^2^ Department of Nutrition School of Public Health Shahid Sadoughi University of Medical Sciences Yazd Iran; ^3^ Department of Clinical Nutrition School of Nutrition and Food Science Food Security Research Center Isfahan University of Medical Sciences Isfahan Iran; ^4^ Department of Nutrition School of Public Health North Khorasan University of Medical Sciences Bojnurd Iran

**Keywords:** anthocyanins, anthropometric indices, body composition, meta‐analysis, randomized controlled trial

## Abstract

**Background and Aims:**

Anthocyanins (ACNs) are water‐soluble plant pigments belong to flavonoids with beneficial effects on health and disease prevention. Some studies have examined the effect of ACNs on anthropometric and body composition indices, but the findings were inconsistent. This systematic review and meta‐analysis aimed to investigate the effect of ACNs and sources rich in anthocyanins on body mass index (BMI), body weight (BW), waist circumference (WC), hip circumference (HC), waist‐hip ratio (WHR), percentage of fat mass (PFM) and fat free mass (FFM).

**Methods:**

PubMed, Web of Science, Scopus, and Google Scholar were searched with no limitation until May 2021 to find relevant randomized controlled clinical trials (RCT). The risk of bias was assessed utilizing Cochrane collaboration's tool. Weighted mean differences (WMD) and 95% confidence intervals (CIs) were obtained using a random effects model.

**Results:**

A total of 31 RCTs (with 0.77–640 mg/day of ACNs supplementation for 28–90 days) with 1438 participants were included. No significant effect was found in BMI, WC, HC, WHR, PFM and FFM after ACNs consumption.

**Conclusions:**

The results showed that ACNs did not significantly affect anthropometric and body composition parameters. Further high‐quality RCTs are required to validate these findings.

## Graphical abstract

This systematic review and meta‐analysis aimed to investigate the effect of Anthocyanins and their rich sources on body mass index, body weight, waist circumference, hip circumference, waist‐hip ratio, percentage of fat mass and fat free mass.

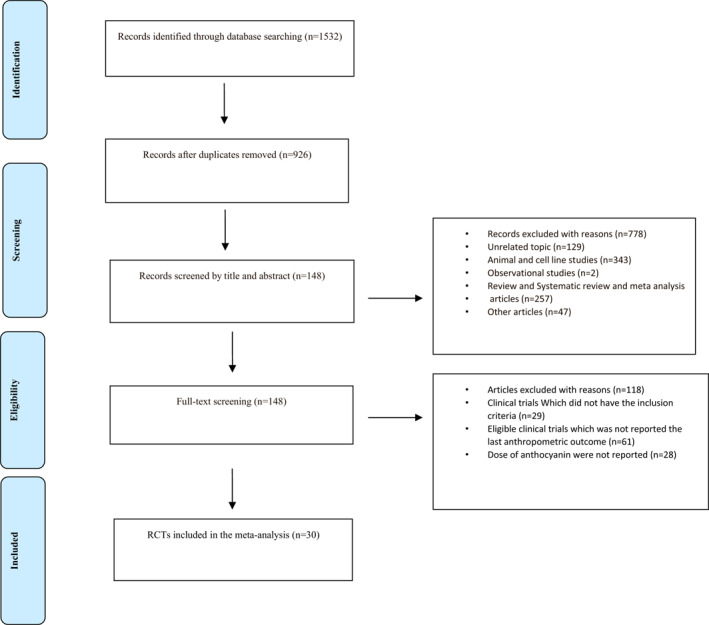

## 1 INTRODUCTION

Anthocyanins (ACNs), a subgroup of polyphenolic compounds, are water‐soluble vacuolar pigments in red, orange, purple and blue plant sources such as fruits, vegetables, grains, cereals and flowers.^1^, ^2^, ^3^, ^4^ ACNs are widely utilized as potential alternatives to synthetic dyes.^3^, ^4^, ^5^ Furthermore, the antioxidant and anti‐inflammatory properties of ACNs, turn them into an attractive material for pharmacological activities.^6^ ACNs alleviate the activity of transcription factor NF‐κB in the cell nucleus by decreasing gene expression of inflammatory cytokines, exerting anti‐inflammatory action.^7^

Therefore, ACNs have an important role in the prevention and treatment of numerous chronic diseases,^8^, ^9^ such as type 2 diabetes mellitus (T2DM),^10^ metabolic syndrome,^11^ dyslipidemia, cardiovascular disease,^12^, ^13^ bone loss in postmenopausal smokers, eye diseases, and cancer.^8^, ^14^ Excessive adipose tissue leads to chronic oxidative stress and inflammation, which is the main reason for morbidities and mortalities in societies.^15^, ^16^, ^17^, ^18^, ^19^, ^20^, ^21^, ^22^, ^23^, ^24^, ^25^, ^26^, ^27^, ^28^, ^29^, ^30^, ^31^, ^32^, ^33^, ^34^, ^35^, ^36^, ^37^, ^38^, ^39^

A recent meta‐analysis of RCT indicated that the ACN supplementation of 300 mg/day or less for 4 weeks is sufficient to reduce the BMI and BW compared to higher‐dose and longer treatments.^40^ However, this meta‐analysis did not demonstrate a significant effect of ACN consumption on other anthropometric indices including waist‐hip ratio (WHR), percentage of fat mass (PFM), hip circumference (HC), and fat‐free mass (FFM).^40^ The authors of this study found some degrees of ambiguity between the results of their study,^40^ and another meta‐analysis conducted by Akhlaghi et al.^41^ According to the results of a study,^41^ ACNs had no significant effects on the anthropometric indices. These results were obtained through a detailed analysis of 5 RCTs that considered only body mass index (BMI) and waist circumference (WC) indices. On the other hand, there were high‐quality RCT studies^15^, ^16^, ^17^, ^20^, ^22^, ^23^, ^24^, ^25^, ^28^, ^29^, ^31^, ^32^, ^36^, ^37^, ^38^, ^39^, ^42^, ^43^, ^44^, ^45^, ^46^, ^47^ that were not included in previous meta‐analysis studies.

Considering the importance of obesity in the occurrence of chronic diseases and the contradictory findings about the effect of anthocyanin on weight and body composition, the current systematic review and meta‐analysis aimed to investigate the effect of ACNs and sources rich in anthocyanins on anthropometric parameters and body composition indices.

## 2 MATERIALS AND METHODS

The current systematic review and meta‐analysis was done based on the Preferred Reporting Items for Systematic Reviews and Meta‐Analyses Guidelines (PRISMA) database.^48^ The study protocol was registered in the international prospective register of systematic reviews (PROSPERO) database (https://www.crd.york.ac.uk/prospero/registrycode=CRD42022315518).

### 2.1 Search strategy

Literature searches were carried out in online databases including PubMed, Web of Science, Scopus, and Google Scholar until May 2022 with no restrictions to find RCTs that have evaluated the effect of ACNs on anthropometric parameters and body composition. The combination of MESH and non‐MESH terms were applied to search as follows: ((anthocyanins OR “anthocyanin extract” OR cyaniding OR pelargonidin OR delphinidin OR peonidin OR petunidin) AND (“Randomized Controlled Trial” AND (weight OR “body mass index” OR obesity OR “hip circumference” OR “waist circumference” OR “body composition indices” OR “body fat” OR “Waist‐Hip Ratio” OR “fat mass” OR “Fat‐free mass”)). Complete search strategy is brought in Supplemental Table 1. Additionally, to make sure the comprehensiveness of searches, references of the included papers were screened for further related studies.

### 2.2 Selection criteria

The patient/population, intervention, comparison, outcome and study type's criteria have been presented in Table 1. The selected studies had the following criteria: (a) the study design was RCT (parallel or cross‐over design); (b) evaluated the effect of pure ACNs and sources rich in anthocyanins on BW, BMI, WC, HC, WHR, PFM and FFM versus placebo/control; (c) were done among adults (age ≥18 years); (d) had sufficient information for the dosage of the ACNs or reported a quantifiable ACNs content for ACNs and sources rich in anthocyanins; (e) had adequate data regarding the mean and standard deviation (SD) or standard error (SE) for baseline and final values or the mean changes of at least one of the outcomes (BW, BMI, WC, HC, WHR, PFM, FFM). Moreover, studies were excluded if they had extra intervention other than pure ACNs and sources rich in anthocyanins like further supplements or herbal products or medications.

**TABLE 1:** The Patient/Population, Intervention, Comparison, Outcome Study types (PICOS) criteria.

Criteria	Description
Population	Adults aged ≥18 years
Intervention	Pure ACNs or products rich in ACNs including extracts, beverages, powders or juices
Comparison	Placebo capsule, control beverage, water
Outcome	BW, BMI, WC, HC, WHR, PFM, FFM
Study types	Randomized controlled clinical trials

### 2.3 Study selection

Two researchers (Faezeh Yarhosseini and Zohreh Sadat Sangsefidi) screened the titles and abstracts of the articles separately to exclude studies that were clearly not relevant. Then, the full texts of all related researches were assessed via the reviewers to select the articles about the effect of ACNs (pure ACNs and sources rich in anthocyanins including extracts, beverages, powders or juices) on anthropometric indices (BMI, WC, HC, WHR), and body composition (PFM, FFM). Finally, any disagreement was resolved by discussion and consulting with other researchers (Mahdieh Hosseinzadeh and Mina Darand).

### 2.4 Data extraction

Data extraction was conducted from the selected trials by the following criteria: authors' family names, year of publication, location of study, number of participants in the intervention and the placebo/control groups, dose and type of intervention, duration of the study, study design, participants sex, age, health status and other characteristics, as well as mean and SD or SE of the anthropometric measurements (BMI, WC, HC, WHR) and body composition (PFM, FFM) at the beginning and end of the study.

### 2.5 Risk of bias assessment

The potential risk of bias of included researches was assessed based on Cochrane Collaboration's tool for systematic reviews of interventions containing the following items: (a) random sequence generation (selection bias); (b) allocation concealment (selection bias); (c) blinding of participants and personnel (performance bias); (d) blinding of outcome assessment (detection bias); (e) incomplete outcome data (attrition bias); (f) selective reporting (reporting bias). The judgment of potential bias depends on the Cochrane Handbook recommendations, which are stratified as low risk of bias, high risk of bias, and uncertain risk of bias.^49^

### 2.6 Data synthesis and analysis

The mean and standard deviation (SD) or standard error (SE) between the intervention and control groups, was used as the effect size for favorable outcomes (BMI, WC, HC, WHR, PFM, FFM). The WMD and its 95% CIs, were calculated by using a random effects model, taking the between‐study variability into account. In the trials in which the SE value was reported, SD was calculated from SE through the following formula: SD = SE×√*n* (*n* = number of participants in each group). Statistical heterogeneity between the studies was examined as follow: *Q* statistic *p* value of <0.1; weak heterogeneity: *I* ^2^ = 25–50, relatively high heterogeneity: *I* ^2^ = 50–75, high heterogeneity: *I* ^2^ = (75–100).^8^, ^50^ Furthermore, the possible sources of heterogeneity were identified by performing subgroup analysis. The criteria for defining subgroups were based on previous studies and heterogeneity among the included studies. Subgroup analyses were based on the participant's health status (healthy subjects/subjects with any disease), assessing the anthropometric measurements as primary or secondary outcome, intervention type (pure ACNs and sources rich in anthocyanins), study location (Asia or others), study design (Parallel or Cross over), study duration and dosage of administered ACNs.

Sensitivity analysis was also conducted to assess the robustness of pooled estimates by sequentially excluding the studies from meta‐analysis and comparing the overall effect. In addition, publication bias was evaluated by using Begg's test with *p*‐value of < 0.05.^50^ All statistical analysis were done using STATA, version 11.2 (Stata Corp). *p*‐value <0.05 was considered statistically significant.

## 3 RESULTS

### 3.1 Literature search

**FIGURE 1:**
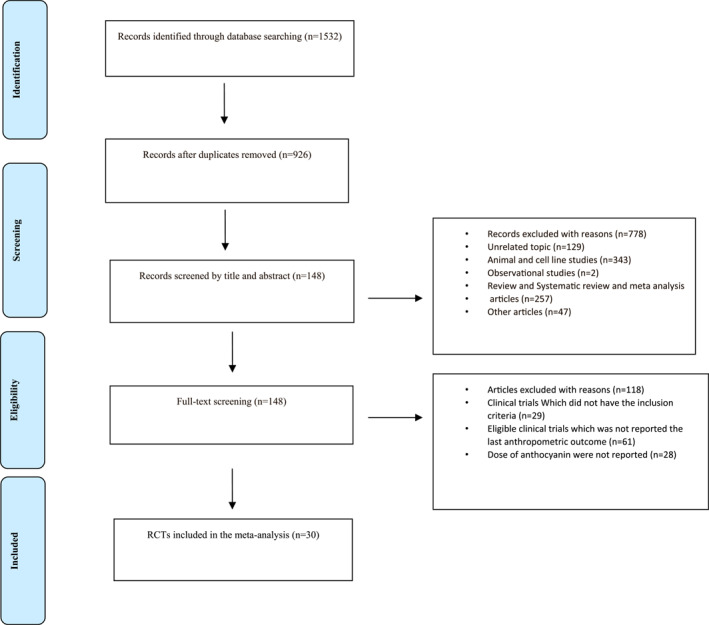
Flow chart for study identification and selection into the meta‐analysis

The systematic search of electronic databases yielded a total of 1532 publications. Then, 30 studies that met the inclusion criteria were included in the present systematic review and meta‐analysis (Figure 1).

### 3.2 Characteristics of the included studies

The main characteristics of all studies that entered this meta‐analysis were indicated in Tables 2 and 3. All studies were published from 2005 to 2022. The total number of participants in these studies was 1438 individuals who were divided into two groups: intervention (*n* 800) and placebo (*n* 638). The countries in which the studies were conducted included (Tokyo, Japan, China, Korea, Taiwan, Iran, India, and Japan) and Non‐Asian countries (Finland, France, Spain, United Kingdom (UK), Australia, United States of America (USA), Canada, Brazil, Denmark, and Italy). All studies had an RCT parallel design, except seven studies that had a cross‐over design.^42^ The duration of trials ranged from 28 to 90 days, and the dose of anthocyanins was from 0.77 to 640 mg/day. In addition, only six studies have evaluated the anthropometric indices and body composition as primary outcomes.

**TABLE 2:** Characteristics of included studies (Anthropometric indices: BW, BMI, WC, HC, WHR).

StudyYear	Country	Number sex (F/M) (P/T)	Age (years)	Design	BMI (kg/m^2^)	Duration (week)	Type of intervention	ACNs dose (mg/day)	Outcomes	Population	Results
Zhu et al. 2011^27^	China	150, *T* = 44/3, *p* = 43/32	40–65	Cross‐over	26.6	12	Berries	320	BW, BMI, WC, HC, WHR	Hypercholesterolemic	**NS**
Soltani et al. 2011^51^	Iran	50, *T* = 25, *p* = 25, 40% male	Age ≥18	Parallel	26.48	6	V.arctostaphs fruit extract	90 ± 4	BMI	Hypercholesterolemic	NS
Thompson et al. 2017^35^	Australia	16, 13F/3M	38 ± 12	Cross‐over	BMI <25	4	Purified ACN	320	BW, BMI	Sedentary/healthy population	NS
Stote et al. 2019^29^	Canada	52, *T* = 26, *p* = 26	51–75	Parallel	26–45	8	Freeze‐dried blueberries	261.8	BW, BMI, WC	Men with type 2 diabetes	NS
Sari et al. 2018^30^	India	25, *T* = 14, *p* = 11	30–55	Parallel	26.48	6	Rosela beverage	81.16	BW, BMI, WC	Obese adult men	NS
Riso et al. 2012^31^	Italy	18	47.8 ± 9.7	Cross‐over	24.8 ± 2.6	6	Wild blueberries	375	BW, BMI	CVD	NS
Qin et al. 2009^33^	China	120, *T* = 39/21 *p* = 39/21	40–65	Parallel	26.1	12	Berry derived ACN	320	BW, BMI, WC	Dyslipidemia	**NS**
Pokimica et al. 2019^34^	Spain	84, F/*M* = 52/32, *T* = 27 AMJ: *p* = 28, AMJd:*p* = 29	40.6 ± 7.1	Parallel	27.08	4	Chokeberry‐juice	AMJ = 113.3 mg/100 ml AMJd = 28.3 mg/100 ml	Women:BW, WC, Men:WC	Healthy	NS
Thompso et al, 2017^35^	Australia	26, 17/9	39 ± 11	Cross‐over	BMI >25	4	Purified ACN	320	BW, BMI	Overweight/obese	NS
Park et al., 2015^36^	Korea	39, *T* = 20, *p* = 19	20–30	Parallel	20 < BMI<25	4	Korean black raspberry	123	BW, WC	Healthy male smokers.	**NS**
Hansen et al. 2005^37^	Denmark	69, red wine = 10/9, half dose = 9/6, full dose = 10/7, placebo = 9/9	38–74	Parallel	25.27	4	1‐Redwine (F/M)	74.9/112.4	BW	Healthy	NS
							2‐Red grape extract‐full dose	67.1/99.1			
							3‐Red grape extract‐half dose	34.3/51.44			
Kusunok et al. 2014^50^	Japan	*T* = 3/4, *p* = 1/10	12–62	Parallel	28.05	8	Black soybean extract	56	BW BMI	Type 2 diabetics	NS
Davinelli et al 2015^38^	Italy	42, *T* = 5/11 *p* = 8/18	45–65	Parallel	25–30	4	Standardized extract of maqui berry	162	BW, BMI WC, HC, WHR	Overweight volunteer smokers	NS
Curtis et al. 2009^39^	East Anglia, Norwich	*T* = 26, *p* = 26	<70 yearsMean: 58.2	Parallel	20–32	12	Elderberry	500	BW	Healthy postmenopausal women	NS
Cardilea et al. 2015^18^	Italy	60	21–50	Parallel	25–35	12	Morosil® extract	340	BW, BMI, WC, HC	Overweight healthy human	Reduction in all variable
Jamar et al. 2020^46^	Brazil	35, 21F/14M, 17P/18T	31–59	Parallel	34.35	6	Juçara pulp powder	130.7	BW, BMI, WC	adults with obesity	NS
Gamel et al. 2020^47^	Canada	28, 14F/14M 15P/13T	18–70	Parallel	BMI ≥25 28.3	8	Bran‐enriched whole or placebo	1.65	BW BMI	Overweight, obese adults, chronic inflammation	NS

Abbreviations: ACN, anthocyanin; F, females; M, males; T, treatment; P, placebo; NS, not significant; B, blinded; R, randomized; BW, body weight; BMI, body mass index; WC, waist circumference; WHR, waist‐hip ratio; CVD, cardiovascular disease; MetS, metabolic syndrome

**TABLE 3:** Characteristics of included studies (body composition indices: PFM, FFM).

StudyYear	Country	Number sex (F/M) (P/T)	Age (years)	Design	BMI (kg/m^2^)	Duration (week)	Type of intervention	ACNs dose (mg/day)	Outcomes	Population	Results
Yamashita et al. 2018^19^	Tokyo	74, 28F/9M, 37 P/37T	30–60	Parallel	21.9	4	Capsules MaquiBright®	41	BW, BMI, PFM	Eye disease	NS
Martin et al. 2019^20^	USA	26, 18F/8M	20–60	Cross‐over	31.3 ± 6.0	4	Tart cherry juice	15.6	BW, BMI, WC, PFM, FFM	Overweight/obese	NS
Maeda‐Yamamoto et al. 2019^21^	Japan	76, 47F/32M, 37P/39T	21–55	Parallel	23	12	“Sunrouge” extract	11.2	BW, PFM	Healthy	NS
Li et al. 2015^22^	China	58, *T* = 12/17 *p* = 12/17	56–67	Parallel	24	24	Purified ACN capsule	320	BW, BMI, PFM	Diabetic patients	NS
Lee et al. 2016^23^	Korea.	63, *T* = 32, *p* = 31	19–65	Parallel	BMI >23	8	Soybean testa extracts	31.45	BMI WC, PFM FFM	Overweight and obese	NS
Dallas et al. 2008^16^	France	20, *p* = 9/1, *T* = 7/3	40–65	Parallel	27–30	12	Citrus‐based polyphenolic dietary supplement	233.8	BW, BMI PFM	Overweight	All variable decrease
Bakuradze et al 2019^52^	Spain	57, *T* = 30, *p* = 27	20–50	Parallel	19–25	8	ACN‐rich fruit juice	274.5	BW, PFM FFM	Healthy males	Increase FFM
Wright et al. 2013^25^	Australia.	16, *T* = 8, *p* = 8	18–65	Parallel	32.8 ± 4.6	4	Sachets dried purple carrot	118.5	BW, BMI WC, PFM FFM	Overweight/obese mens	NS
Chang et al. 2014^17^	Taiwan	36, *T* = 7/12, *p* = 8/9	18–65	Parallel	BMI >27	12	Hibiscus sabdariffa extracts	25	BW, BMI WC, PFM, HC, WHR	Overweight/obese	WC, PFM and WHR decrease
Kima et al. 2018^26^	USA	37, *p* = 8, *T* = 19, 26/11	18–65	Parallel	33.5 ± 6.7	12	açaí‐beverage	199.55	BW BMI WC PFM HC	Metabolic syndrome	NS
Yarahmadi et al. 2014^32^	Iran	22/32, *p* = 17, *T* = 27	23.89	Parallel	22.96	6	Purified ACN	100	BW, PFM	athletes	NS
Sarah et al. 2020^40^	USA	19, 9F/10M, 10P/9T	20–60	Parallel	21.9	12	Tart cherry juice	88	BW, BMI WC, PFM, FFM, HC, WHC	MetS	**NS**
Kimble et al. 2021^45^	UK	52, 16F/34M, 26P/26T	20–60	Parallel	31.3 ± 6.0	12	Montmorency cherries	70.5	BW, BMI, PFM, FFM, WC	25<(BMI)>40	NS

Abbreviations: ACN, anthocyanin; F, females; M, males; T, treatment; P, placebo; NS, not significant; B, blinded; R, randomized; BW, body weight; BMI, body mass index; WC, waist circumference; WHR, waist‐hip ratio; CVD, cardiovascular disease; MetS, metabolic syndrome

### 3.3 Risk of bias assessment

The risk of bias assessment of the included studies has been shown in Table 4. Even though the eligible studies were randomized, sufficient information was not presented in 17 studies for random sequence generation and 5 studies for allocation concealment.^19^, ^21^, ^35^, ^38^, ^53^ Furthermore, 9 trials had blind participants and personnel. The risk of detection bias (blinding of outcome assessment) was also unclear among all surveys except for six trials.^15^, ^17^, ^18^, ^19^, ^21^, ^35^ The risk of attrition bias (incomplete outcome data) was also low among all surveys except in one trial which was unclear.^44^ Reporting bias (selective reporting) and other potential sources of bias were low in all included studies.

**TABLE 4:** Study quality and risk of bias assessment using Cochrane Collaboration's tool.

Author (Year)	Random sequence generation	Allocation concealment	Blinding of participants and personnel	Blinding of outcome assessment	Attrition bias	Selective reporting
Yamashita et al. 2018^19^	Low	Unclear	Low	Low	Low	Low
Martin et al. 2019^20^	Low	Unclear	Unclear	Unclear	Low	Low
Mari Maeda‐Yamamoto et al. 2019^21^	Low	Unclear	Low	Low	Low	Low
Li et al. 2015^22^	Low	Unclear	Low	Low	Low	Low
Lee et al. 2016.^23^	Low	Low	Low	Low	Low	Low
Dallas et al. 2008^16^	Unclear	Unclear	Unclear	Unclear	Low	Low
Bakuradze et al. 2019^52^	Low	Low	Unclear	Unclear	Low	Low
Wright et al. 2013^25^	Low	Low	Low	Low	Low	Low
Chang et al. 2014^17^	Unclear	Unclear	Unclear	Unclear	Low	Low
Kima et al. 2018^26^	Unclear	Unclear	Unclear	Unclear	Low	Low
Curtis et al. 2009^39^	High	Low	Low	Low	Low	Low
Cardilea et al. 2015^18^	Unclear	Unclear	Unclear	Unclear	Unclear	Low
Thompso et al. 2017^35^	Low	Unclear	Low	Unclear	Low	Low
Zhu et al. 2011^27^	Unclear	Unclear	Unclear	Unclear	Low	Low
Yarahmadi et al. 2014^32^	Low	Unclear	Unclear	Unclear	Low	Low
Jamar et al. 2020^46^	Low	Low	Low	High	Low	Low
Sarah et al. 2020^40^	Low	Unclear	High	High	Low	Low
Kimble et al. 2021^45^	Low	Unclear	Low	Unclear	Low	Low
Stote et al. 2019^29^	Low	Unclear	Low	Unclear	Low	Low
Pokimica et al. 2019^34^	Low	Unclear	High	Low	Low	Unclear

### 3.4 Meta‐analysis

#### 3.4.1 Effect of ACNs on body composition (PFM, FFM)

**FIGURE 2:**
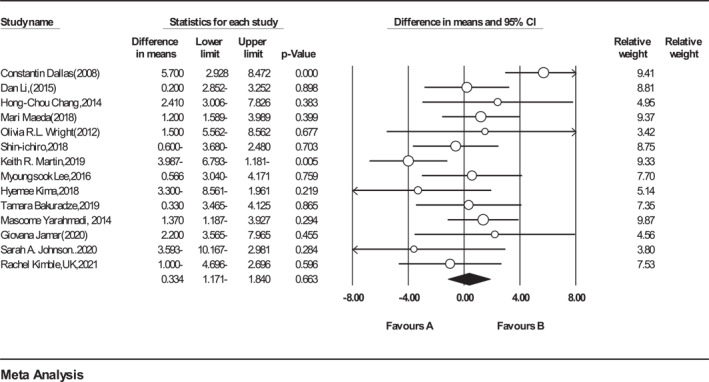
Forest plot detailing weighted mean difference and 95% confidence intervals (CIs) for the effect of Anthocyanins supplementation on percentage of fat mass.

**FIGURE 3:**
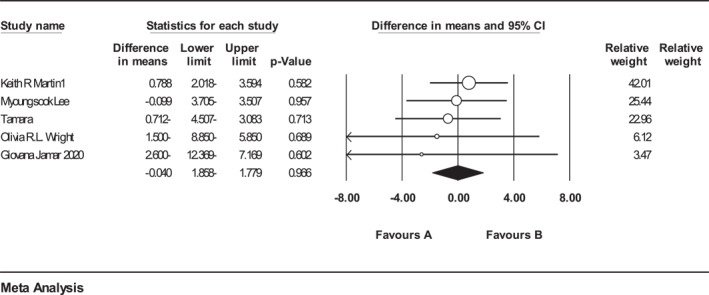
Forest plot detailing weighted mean difference and 95% confidence intervals (CIs) for the effect of Anthocyanins supplementation on fat free mass.

Meta‐analysis on 14 eligible studies (*n* = 631, intervention: *n* = 329, placebo: *n* = 310) that presented data on PFM showed that no significant difference was observed in PFM between intervention and control groups (Figure 2). (WMD = 0.33%, 95% CI = −1.17 to 1.84, *p* = 0.66). A weak heterogeneity was detected among the included trials (*Q*‐value = 46.89, Cochrane *Q* test, *p* < 0.001; *I* ^2^ = 48.82) and also based on the results of the analysis of 5 included studies on FFM reported that (*n* = 223, intervention: *n* = 114, placebo: *n* = 109), intake of ACNs had no significant impact on FFM (Figure 3). (WMD = −0.040%; 95% CI: −1.85, 1.77%; *p* = 0.96). There was no heterogeneity between studies (*Q* statistic = 0.87, *I* ^2^ = *p* < 0.001, *p* = 0.92).

#### 3.4.2 Effect of ACNs on anthropometric indices (BMI, WC, HC, WHR)

**FIGURE 4:**
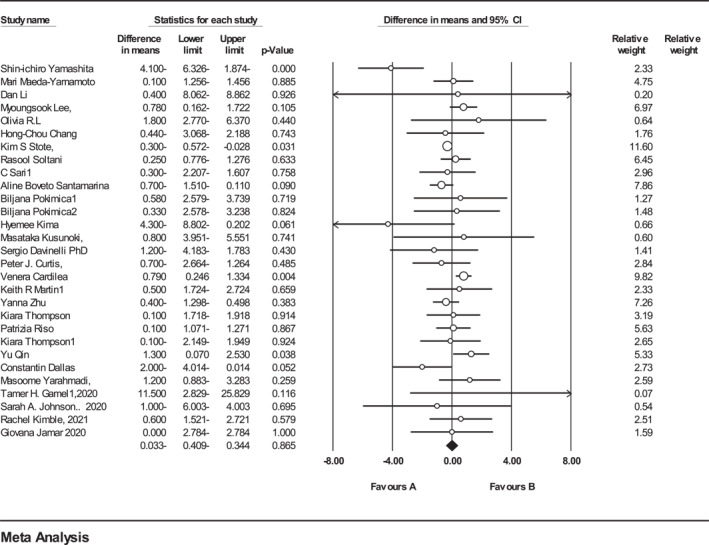
Forest plot detailing weighted mean difference and 95% confidence intervals (CIs) for the effect of Anthocyanins supplementation on Body Mass Index.

**FIGURE 5:**
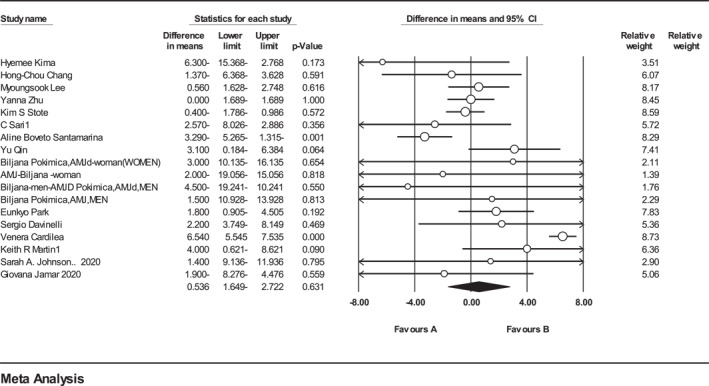
Forest plot detailing weighted mean difference and 95% confidence intervals (CIs) for the effect of Anthocyanins supplementation on waist circumference.

**FIGURE 6:**
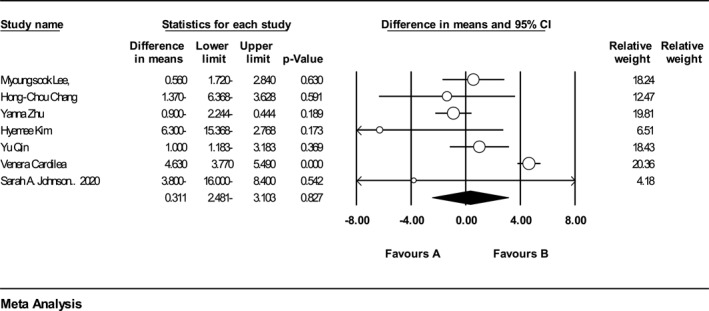
Forest plot detailing weighted mean difference and 95% confidence intervals (CIs) for the effect of Anthocyanins supplementation on hip circumference.

**FIGURE 7:**
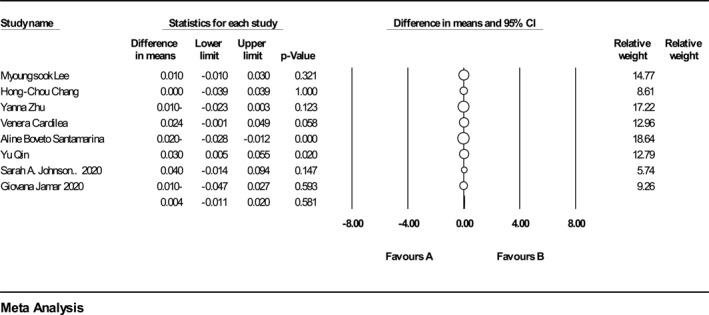
Forest plot detailing weighted mean difference and 95% confidence intervals (CIs) for the effect of Anthocyanins supplementation on waist‐hip ratio.

29 trials (*n* = 631, intervention: *n* = 329, placebo: *n* = 310) reported the effect of ACN on BMI. The analysis indicated that ACNs supplementation had no significant effect on BMI (WMD = 0.033 kg/m^2^, 95% CI = −0.409 to 0.344, *p* = 0.86) (Figure 4). A poor heterogeneity was observed among the included trials (*Q*‐value = 49.93, Cochrane *Q* test, *p* < 0.001; *I* ^2^ = 43.92). Pooled analysis of 18 RCTs (440 in the intervention group and 426 in the placebo group) did not demonstrate any significant effect of ACNs supplementation on WC (Figure 5). (WMD = 0.53 cm; 95% CI: −1.64, 2.72 cm; *p* = 0.63). There was high heterogeneity between the included studies (*Q* statistic = 136.90, *I* ^2^ = 87.58, *p* < 0.001). The analysis of 7 trials (*n* = 476, intervention: *n* = 239, placebo: *n* = 237) could not reveal a significant effect on the HC after intake of ACNs (Figure 6). (WMD = 0.31 cm; 95% CI: 2.48‐, 3.10 cm; *p* = 0.82). There was high heterogeneity between studies (*Q* statistic = 59.67, *I* ^2^ = 89.94, *p* < 0.001) and also the results of 8 eligible studies (*n* = 506, intervention: *n* = 25, placebo: *n* = 25) showed that consuming ACNs had no significant effect on WHR (Figure 7). (WMD = 0.00 cm; 95% CI: 0.02, 0.04 cm; *p* = 0.65). There was high heterogeneity between the studies (*Q* statistic = 132.01, *I* ^2^ = 94.69, *p* < 0.001).

### 3.5 Sensitivity analysis and publication bias

Sensitivity analysis indicated that the results of the present meta‐analysis were not dependent on the exclusion of a particular study. Statistical asymmetry test (Begg's tests) did not indicate the presence of publication bias for the eligible studies related to BMI (*p* = 0.92), FM (*p* = 0.41), WC (*p* = 0.30), WHC (*p* = 0.21) and HC (*p* = 1.00). However, Begg's test showed a significant publication bias for the studies regarding FFM (Begg's test, *p* = 0.02). After adjusting the effect size for potential publication bias by the “trim and fill” correction, 2 missing studies were needed in the funnel plot that brings the *p*‐value to >0.05 (Adjusted values: WMD = 0.15%, 95% CI = −1.57, 1.89).

#### 3.5.1 Subgroup analysis

No significant impacts on BMI, WC, HC, WHR, PFM, and FFM was found in all subgroup analysis following ACNs supplementation. The detailed results of the subgroup analysis are presented in Table 5.

**TABLE 5:** Subgroup analysis of included randomized controlled trials in meta‐analysis of the effect of ACNs supplementation on Anthropometric Parameters and Body Compositions.

Group	Number of trials	WMD (95% CI)	*p* value*	*Q* statistics (*p*)	*I* ^2^ (%)	*P* for between subgroup heterogeneity
Fat mass (%) Health status						
Subjects with any disease	5	−0.529 (−2.337 1.278)	0.566	3.253	0.0	
Healthy	9	0.814 (−1.236, 2.864)	0.437	24.699	67.610	0.002
Country						
Asian	6	0.740 (−0.546, 2.025)	0.259	1.559	0	0.547
Non‐Asian	8	−0.181 (−3.163, 2.802)	0.905	27.658	84.27	
Dosage of ACNs						
160 mg/day or lower	11	−0.332 (−1.709, 1.045)	0.637	13.923	28.178	0.008
More than 160 mg/day	3	2.190 (−1.609, 5.989).	0.259	8.553	76.616	
Duration						
≤4 weeks	4	−0.847 (−3.544, 1.851)	0.539	7.290	58.846	0.082
6–8 weeks	4	1.039 (−0.704, 2.781)	0.243	13.4	0.0	
12 weeks ≤	6	0.487 (−2.601, 3.575)	0.757	16.875	70.370	
Intervention type						0.804
Pure ACN	4	0.459 (−1.195, 2.112)	0.587	0.969	0.0	
Non pure ACN	10	0.578 (−2.005, 3.162)	0.661	26.037	3.115	
Type of studies in terms of assessing anthropometric indices						0.905
Studies evaluated anthropometric indices as primary outcome	3	0.332 (−3.407, 4.071)	0.862	2.799	28.544	
Studies evaluated anthropometric indices as secondary outcomes	11	0.324 (−1.369, 2.016)	0.708	2.799	62.640	
Design						
Cross over	1	−3.987 (−6.793,−1.181)	0.005	0.0	0.0	0.001
Parallel	13	0.870 (−0.442, 2.183)	0.194	18.627	35.577	
Overall	**14**	**0.334** (−**1.171, 0.663)**	**p = 0.663**	**27.06**	**63.056**	
Body mass index (Kg/m^2^)						
Type of studies in terms of assessing anthropometric indices						
Studies evaluated anthropometric indices as primary outcome	7	0.174 (−0.698, 1.046)	0.696	9.021	33.488	0.005
Studies evaluated anthropometric indices as secondary outcomes	22	−0.103 (−0.505, 0.299)	0.615	33.136	36.624	
Health status						
Subjects with any disease	13	−0.404 (−1.077, 0.295)	0.256	27.952	53.574	0.005
Healthy	16	0.322 (−0.015, 0.659)	0.061	13.883	0.0	
Intervention type						
Pure ACN	6	0.297 (−0.400, 0.993)	0.404	5.753	13.082	0.277
Non pure ACN	23	−0.145 (−0.587, 0.297)	0.520	43.000	48.838	
Dosage of ACNs						
≤ 130 mg/day	11	0.165 (−0.464, 0.793)	0.607	27.297	34.59	0.054
> 130 mg/day	18	−0.206 (−0.664, 0.251)	0.377	18.932	52.461	
Duration						
≤ 4 weeks	9	−0.364 (−1.391, 0.662)	0.486	13.883	42.374	0.051
6–8 weeks	9	−0.036 (−0.494, 0.421)	0.876	11.370	29.638	
12 weeks ≤	11	0.003 (−0.668, 0.674)	0.993	49.936	46.655	
Design						
Cross over	5	−0.116 (−0.724, 0.491)	0.707	0.864	0.0	0.897
Parallel	24	−0.051 (−0.518, 0.416)	0.831	49.054	57.704	
Country						
Asian	6	0.297 (−0.400, 0.993)	0.404	5.753	13.82	0.277
Non‐Asian	23	−0.145 (−0.587, 0.297)	0.520	43.000	48.838	
Overall	**29**	**−0.056** (−**0.449, 0.337)**	**0.779**	**46.896**	**48.823**	
Waist circumference (cm)						
Health status						
Subjects with any disease	7	−0.045 (−1.031, 0.941)	0.928	5.998	0.000	0.000
Healthy	11	0.960 (−2.285, 4.205)	0.562	98.701	89.868	
Intervention type						
Pure ACN	2	1.217 (−1.750, 4.184)	0.421	2.707	63.055	0.025
Non pure ACN	16	0.320 (−2.245, 2.885)	0.807	129.177	88.388	
Dosage of ACNs						
≤ 130 mg/day	11	0.679 (−0.725, 2.083)	0.343	7.662	0.000	0.017
> 130 mg/day	7	0.975 (−2.477, 4.426)	0.580	123.564	95.144	
Design						
Cross over	2	1.398 (−2.340, 5.136)	0.464	0.978	2.539	0.019
Parallel	16	0.289 (−2.230, 2.809)	0.822	128.903	88.363	
Country						
Asian	7	0.599 (−0.472, 1.670)	0.273	5.384	0.000	0.000
Non‐Asian	11	0.424 (−3.183, 4.031)	0.818	118.679	91.574	
Type of studies in terms of assessing anthropometric indices						
Studies evaluated anthropometric indices as primary outcome	4	0.565 (−5.348, 6.477)	0.852	87.841	0.000	0.000
Studies evaluated anthropometric indices as secondary outcomes	14	0.213 (−1.051, 1.477)	0.741	41.550	0.052	
Overall	**18**	**0.536 (1.649, 2.722)**	**0.631**	**136.905**	**87.583**	
Hip circumference (cm)						
Health status						
Subjects with any disease	2	−0.467 (−1.970, 1.037)	0.543	3.841	21.899	0.000
Healthy	5	2.190 (−1.666, 6.046)	0.266	12.327	83.775	
Intervention type						
Pure ACN	2	0.153 (−1.972, 1.666)	0.869	2.110	63.055	0.000
Non pure ACN	5	0.435 (−3.212, 4.082)	0.815	21.665	88.388	
Dosage of ACNs						
≤ 130 mg/day	4	−0.594 (−1.717, 0.529)	0.300	1.541	0.000	0.000
> 130 mg/day	3	1.710 (−2.160, 5.580)	0.387	14.248	85.963	
Country						
Asian	3	0.990 (−1.922, 3.903)	0.505	54.991	92.726	0.032
Non‐Asian	4	−5.410 (−12.688, 1.867)	0.145	0.104	0.000	
Type of studies in terms of assessing anthropometric indices						
Studies evaluated anthropometric indices as primary outcome	2	2.156 (−3.633, 7.945)	0.465	5.377	81.402	0.000
Studies evaluated anthropometric indices as secondary outcomes	5	0.216 (1.428, 0.996)	0.727	4.672	14.383	
Overall	**7**	**0.311 (2.481, 3.103)**	**0.827**	**59.671**	**89.945**	
Hip circumference						
Health status						
Subjects with any disease	5	0.009 (−0.009, 0.028)	0.316	10.664	62.489	0.002
Healthy	3	−0.003 (−0.033, 0.027)	0.847	11.173	82.099	
Intervention type						
Pure ACN	2	0.010 (−0.023, 0.043)	0.559	2.263	55.805	0.024
Non pure ACN	6	0.003 (−0.014, 0.020)	0.770	23.622	78.833	
Dosage of ACNs						
< 160 mg/day	5	−0.002 (−0.022, 0.018)	0.865	12.435	67.834	0.006
≥ 160 mg/day‬‬‬‬‬‬‬‬‬‬‬‬‬‬‬‬‬‬‬‬‬‬‬‬‬‬‬‬‬‬‬‬‬‬‬‬‬‬‬‬‬‬‬‬‬‬‬‬‬‬‬‬‬‬‬‬‬‬‬‬‬‬	3	0.013 (−0.015, 0.041)	0.367	11.136	82.040	
Country						
Asian	3	0.006 (−0.022, 0.033)	0.679	7.673	73.934	0.107
Non‐Asian	5	0.005 (−0.018, 0.028)	0.686	20.733	80.707	
Type of studies in terms of assessing anthropometric indices						
Studies evaluated anthropometric indices as primary outcome	2	0.008 (−0.031, 0.048)	0.559	7.664	86.952	0.135
Studies evaluated anthropometric indices as secondary outcomes	6	0.004 (−0.017, 0.024)	0.770	21.098	76.301	
Duration						
< 12 weeks	3	−0.008 (−0.030, 0.014)	0.497	7.834	74.469	0.001
12 weeks≤	5	0.013 (−0.008, 0.035)	0.222	12.950	69.112	
Overall	**8**	**0.004 (−0.011, 0.020)**	**0.581**	**30.999**	**77.419**	
Fat free mass (%)						
Health status						
Subjects with any disease	2	0.530 (−2.167, 3.227)	0.700	0.427	0.000	0.575
Healthy	3	0.514 (2.977, 1.949)	0.682	0.130	0.000	
Duration						
≤ 4 weeks	2	0.497 (−2.124, 3.118)	0.710	0.325	74.469	0.578
4 weeks<	3	0.537 (−3.063, 1.988)	0.677	0.236	96.640	
Dosage of ACNs						
< 130 mg/day	2	0.453 (−1.761, 2.668)	0.688	0.145	0.000	0.444
≥ 130 mg/day‬‬‬‬‬‬‬‬‬‬‬‬‬‬‬‬‬‬‬‬‬‬‬‬‬‬‬‬‬‬‬‬‬‬‬‬‬‬‬‬‬‬‬‬‬‬‬‬‬‬‬‬‬‬‬‬‬‬‬‬‬‬	3	−1.061 (−4.249, 2.127)	0.514	0.142	0.000	
Country						
Asian	2	0.453 (−1.761, 2.668)	0.688	0.145	0.000	0.444
Non‐Asian	3	−1.061 (−4.249, 2.127)	0.514	0.142	0.000	
Overall	5	**0.040 (−1.858, 1.779)**	**0.966**	**0.871**	**0.000**	

*Note*: The meaning of the bold values are overall effects of each variables.

Abbreviations: ACN, anthocyanin; CI, confidence interval; WMD, weighted mean difference.

*p value < 0.05 considered as significant statistical level.

## 4 DISCUSSION

The present study was a comprehensive meta‐analysis that investigated the effect of ACNs supplementation on anthropometric parameters and body composition indices in adults. The overall results of the analyses showed that ACNs supplementation did not significantly affect the anthropometric parameters and body composition indices. In addition, stratification of the studies by dose, the country where the studies were carried out and intervention type (pure ACNs and their rich sources) did not affect the indicators studied.

The results of a meta‐analysis showed that taking ACNs‐containing products (berries, red grapes, red wine) reduced WC but its *p*‐value was not mentioned in the article,^54^ so it is not possible to comment clearly on the significance of this association. The results of the meta‐analysis of Park et al. also showed that in general ACNs supplementation caused a significant reduction in BMI while it did not affect BW and WC.^40^ In the meta‐analysis of García‐Conesa et al.^54^ the beneficial effect of ACNs on WC was observed only in the overweight/obese subgroup. In contrast, in another meta‐analysis, ACNs supplementation significantly reduced BMI only in doses less than 300 mg and the duration of fewer than 4 weeks in the non‐obese. In addition, in this study, ACNs decreased BMI only in the Middle East compared to other countries.^40^ The interpretation of the above results was complex and far from understood. Different factors such as differences in dose and bioavailability of these substances in the gut,^55^, ^56^ various health statuses, ethnicity, and lifestyle of people^41^, ^51^ may be involved in observing these results. In addition to the factors examined in the present meta‐analysis, other important factors may have a critical impact on the association and explain the lack of consistent evidence in humans regarding the weight‐reducing effect of ACNs. One of these factors is the genetic characteristics of host individuals and their gut microbiome and its function.^56^ In other words, scientific evidence suggests that individuals have a different genotype‐dependent response to dietary ingredients.^52^ In recent years, different genetic variants have been identified regarding the interaction of dietary components with individuals' genotypes and their association with the prevalence of obesity.^57^ Another major factor is the bioavailability of dietary components and their metabolites.^58^

Although ACNs did not reduce weight in the present study, their anti‐obesity properties can be attributed to the ability to control food intake,^59^ increase energy expenditure,^60^ improve the inflammatory response,^61^ and inhibit lipid absorption.^62^ Anthocyanins act as the prebiotics that changes the composition of the gut microbiota. This action is related to restoring tight‐junction protein distribution and localization.^63^ Hence, the gut permeability and plasma lipopolysaccharide (LPS) levels (metabolic endotoxemia) are reduced and improve low‐grade inflammation characterizing obesity.^64^ In addition, anthocyanins can boost the growth of Bifidobacterium spp., which increases the intestinal production of a fasting‐induced adipose factor that inhibits fat storage in the host.^65^ These compounds also change the plasma gut peptides levels (enhanced GLP‐1 and PYY, and reduced ghrelin). These actions are associated with satiety, weight loss, and increased insulin sensitivity.^66^

The present study had strengths and limitations. First, the present study was the largest meta‐analysis of RCTs to review systematically the effect of ACNs supplementation on anthropometric parameters and body compositions in adults. Second, the large sample size increases statistical power and generalizability. Third, there was no time limit for eligible studies. Although the heterogeneity between studies was low, there were differences in the design of eligible trials in terms of the participants' characteristics (the health conditions, ethnicity, and lifestyles of people), prescription of pure ACNs, or sources rich in ACNs, dose and duration of supplementation. Furthermore, possible minor effects of other bioactive compounds in ACNs‐rich sources such as polyphenols, flavonoids or other phytochemicals cannot be ignored, so the net effect of ACNs cannot be determined with certainty. Another limitation was the lack of bioavailability assessment among different populations in the world.

## 5 CONCLUSION

In general, the results of the analysis demonstrated that ACNs did not improve anthropometric parameters and body compositions in adults. Further high‐quality clinical trial studies are needed in the future to firmly establish the clinical efficacy of ACNs and find the optimal dose and duration of the intervention in people with various health and ethnic conditions.

**Abbreviations**
ACNs: anthocyanins
BMI: body mass index
BW: body weight
CI: confidence interval
FFM: fat free mass
GLP‐1: Glucagon‐like peptide‐1
HC: hip circumference
NF‐jB: nuclear factor‐kappaB
PFM: percentage of fat mass
PYY: Peptide YY
PRISMA: reviews and meta‐analyses guidelines
RCT: randomized controlled clinical trials
SD: standard deviation
SE: standard error
T2DM: type 2 diabetes mellitus
WC: waist circumference
WHR: waist‐hip ratio
WMD: weighted mean difference

## AUTHOR CONTRIBUTIONS

We are grateful to check our extracted data and contributed to the meta‐analysis. The authors' responsibilities were as follows: —M.H and F.Y designed the research; M.H and F.Y wrote the protocol and conducted the electronic searches; Z.S, M.D, and F.Y independently selected the study and extracted relevant articles and tabulated data; M.H, Z.S, and F.Y analyzed the data and interpretation of results; M.H, F.Y, H.M and M.D wrote the first draft of the manuscript; and all authors read and approved the final version.

## CONFLICT OF INTEREST

The authors declare no conflict of interest to report regarding this study.

## AVAILABILITY OF DATA AND MATERIALS

Not applicable.

## CONSENT FOR PUBLICATION

Not applicable.

## Supporting information

Supporting Information S1
