# Supplementary material for: Does anthocyanins consumption affect weight and body composition? A systematic review and meta‐analysis of randomized controlled trials
Source: Obes Sci Pract. 2023 Jan 23;9(1):42–58. doi: 10.1002/osp4.651 (PMC9913187; doi:10.1002/osp4.651)
Supplement: Supplementary file 1 — Supporting Information S1 [file OSP4-9-42-s001.docx]

((anthocyanins[MeSH] OR anthocyanins[tiab] OR "anthocyanin extract"[tiab] OR cyanidin[Supplementary Concept] OR cyaniding[tiab] OR pelargonidin [Supplementary Concept] OR pelargonidin[tiab] OR delphinidin[Supplementary Concept] OR delphinidin[tiab] OR peonidin[Supplementary Concept] OR peonidin[tiab] OR petunidin[Supplementary Concept] OR petunidin[tiab] ) AND (RCT[tiab] OR “Clinical Trial”[tiab] OR "Randomized Controlled Trial"[tiab] OR "[Randomized Controlled Trials as Topic](https://www.ncbi.nlm.nih.gov/mesh/68016032)"[MeSH] OR random*[tiab] OR intervention*[tiab] OR trial*[tiab] OR Placebos[MeSH] OR Placebos [tiab] OR assignment[tiab] OR cross-over[tiab] OR parallel[tiab] OR randomized[tiab] OR "Clinical Trials as Topic"[MeSH] OR "Clinical Trials as Topic"[tiab] ) AND (weight [tiab] OR overweight[MeSH] OR overweight[tiab] OR "body mass index"[MeSH] OR "body mass index"[tiab] OR BMI[tiab] OR obesity[MeSH] OR obesity[tiab] OR "hip circumference"[tiab] OR "waist circumference"[MeSH] OR "waist circumference"[tiab] OR "body composition indices"[tiab] OR obese[tiab] OR "body fat accumulation"[tiab] OR "lean body mass"[tiab] OR LBM[tiab] OR "body fat"[tiab] OR "Waist-Hip Ratio"[MeSH] OR "[Adipose Tissue](https://www.ncbi.nlm.nih.gov/mesh/68000273)"[tiab] OR "[Adipose Tissue](https://www.ncbi.nlm.nih.gov/mesh/68000273)"[MeSH] OR "Waist-Hip Ratio"[tiab] OR WHR[tiab] OR Anthropometry[MeSH] OR Anthropometry[tiab] OR "[Subcutaneous Fat, Abdominal](https://www.ncbi.nlm.nih.gov/mesh/68050507)"[MeSH] OR "[Subcutaneous Fat, Abdominal](https://www.ncbi.nlm.nih.gov/mesh/68050507)" [tiab] OR "Intra-Abdominal Fat"[MeSH] OR "Intra-Abdominal Fat"[tiab] OR "fat mass"[tiab] OR FM[tiab] OR "abdominal fat"[MeSH] OR "abdominal fat"[tiab] OR adipose[tiab] OR "[Adipose Tissue, Brown](https://www.ncbi.nlm.nih.gov/mesh/68002001)"[MeSH] OR "[Adipose Tissue, Brown](https://www.ncbi.nlm.nih.gov/mesh/68002001)" [tiab] OR "[Adipose Tissue, White](https://www.ncbi.nlm.nih.gov/mesh/68052436)"[MeSH] OR "[Adipose Tissue, White](https://www.ncbi.nlm.nih.gov/mesh/68052436)"[tiab] OR "Fat-free mass"[tiab] OR FFM[tiab] OR "anthropometric measures"[tiab] OR "Visceral fat"[tiab] OR "Body Fat Distribution"[MeSH] OR "Body Fat Distribution"[tiab] OR "Body weight"[MeSH] OR "Body weight"[tiab] OR "Soft lean mass"[tiab] OR "total body water"[tiab] OR BFM[tiab] OR [Adiposity](https://www.ncbi.nlm.nih.gov/mesh/68050154)[MeSH] OR [Adiposity](https://www.ncbi.nlm.nih.gov/mesh/68050154) [tiab] OR "[Body Constitution](https://www.ncbi.nlm.nih.gov/mesh/68001824)" [MeSH] OR "[Body Constitution](https://www.ncbi.nlm.nih.gov/mesh/68001824)"[tiab] OR "Adipose Tissue"[MeSH] OR "Adipose Tissue"[tiab] OR "[Body Weight Changes](https://www.ncbi.nlm.nih.gov/mesh/68001836)"[MeSH] OR "[Body Weight Changes](https://www.ncbi.nlm.nih.gov/mesh/68001836)"[tiab] OR "[Weight Reduction Programs](https://www.ncbi.nlm.nih.gov/mesh/68061217)"[MeSH] OR "[Weight Reduction Programs](https://www.ncbi.nlm.nih.gov/mesh/68061217)"[tiab] OR "Weights and Measures"[MeSH] OR "Weights and Measures"[tiab] OR "[Subcutaneous Fat](https://www.ncbi.nlm.nih.gov/mesh/68050151)"[MeSH] OR "[Subcutaneous Fat](https://www.ncbi.nlm.nih.gov/mesh/68050151)"[tiab]))
